# Supplementary material for: Asynchronous mouse embryo polarization leads to heterogeneity in cell fate specification
Source: eLife. 2025 Dec 8;13:RP101140. doi: 10.7554/eLife.101140 (PMC12685296; doi:10.7554/eLife.101140)
Supplement: Peer review history

# Editor report: eLife Assessment

**DOI:** 10.7554/eLife.101140.3.sa0

**Authors:** Mark S Sharpley

**Affiliations:** Cedars-Sinai Medical Center United States

**Keywords:** Compelling, Fundamental

This **fundamental** work substantially advances our understanding of the molecular basis by which early symmetry breaking events connect to the following cell fate specifications in preimplantation mammalian embryos. The evidence supporting the conclusions is **compelling**, with advanced image based assays and microinjection based functional tests. The work will be of broad interest to cell and developmental biologists.

# Referee report: Reviewer #1 (Public review)

**DOI:** 10.7554/eLife.101140.3.sa1

Summary:

This work starts with the observation that embryo polarization is asynchronous starting at the early 8-cell stage, with early polarizing cells being biased towards producing the trophectoderm (TE) lineage. They further found that reduced CARM1 activity and upregulation of its substrate BAF155 promote early polarization and TE specification, this piece of evidence connects the previous finding that at Carm1 heterogeneity 4-cell stage guide later cell lineages - the higher Carm1-expressing blastomeres are biased towards ICM lineage. Thus, this work provides a link between asymmetries at the 4-cell stage and polarization at the 8-cell stage, providing a cohesive explanation regarding the first lineage allocation in mouse embryos.

Strengths:

In addition to what has been put in the summary, the advanced 3D image-based analysis has found that early polarization is associated with a change in cell geometry in blastomeres, regarding the ratio of the long axis to the short axis. This is considered a new observation that has not been identified.

Weaknesses:

For the microinjection-based method to overexpression/deletion of proteins, although it has been shown to be effective in the early embryo settings and has been widely used, it may not fully represent the in vivo situation in some cases, compared to other strategies such as the use of knock-in mice.

This is a minor weakness and has been discussed by the author in the revised manuscript.

# Author comment: Author response

**DOI:** 10.7554/eLife.101140.3.sa2

**Authors:** Adiyant Lamba, Meng Zhu, Maciej Meglicki, Sylwia Czukiewska, Lakshmi Balasubramaniam, Ron Hadas, Nina Weishaupt, Ekta Patel, Yu Hua Kavanagh, Ran Wang, Naihe Jing, Magdalena Zernicka-Goetz

**Affiliations:** University of Cambridge Cambridge United Kingdom; Harvard Medical School Boston United States; University of Cambridge Cambridge United Kingdom; Leiden University Medical Center Leiden Netherlands; University of Cambridge Cambridge United Kingdom; California Institute of Technology Pasadena United States; University of Cambridge Cambridge United Kingdom; California Institute of Technology pasadena United States; University of Cambridge Cambridge United Kingdom; Chinese Academy of Sciences Shanghai China; Chinese Academy of Sciences Shanghai China; California Institute of Technology Los Angeles United States

The following is the authors’ response to the original reviews.

**Reviewer #1 (Public review):**

Summary:

This work starts with the observation that embryo polarization is asynchronous starting at the early 8-cell stage, with early polarizing cells being biased towards producing the trophectoderm (TE) lineage. They further found that reduced CARM1 activity and upregulation of its substrate BAF155 promote early polarization and TE specification, this piece of evidence connects the previous finding that at Carm1 heterogeneity 4-cell stage guide later cell lineages - the higher Carm1-expressing blastomeres are biased towards ICM lineage. Thus, this work provides a link between asymmetries at the 4-cell stage and polarization at the 8-cell stage, providing a cohesive explanation regarding the first lineage allocation in mouse embryos.

Strengths:

In addition to what has been put in the summary, the advanced 3D image-based analysis has found that early polarization is associated with a change in cell geometry in blastomeres, regarding the ratio of the long axis to the short axis. This is considered a new observation that has not been identified.

Weaknesses:

For the microinjection-based method to overexpression/deletion of proteins, although it has been shown to be effective in the early embryo settings and has been widely used, it may not fully represent the in vivo situation in some cases, compared to other strategies such as the use of knock-in mice. This is a minor weakness; it would be good to include some sentences in the discussion on the potential caveats.

We thank the reviewer for their insightful summary of our work, and their adjudication on the novelty of our research. We agree with the reviewer that microinjection-based methods, whilst being the standard and widely used in the field, have their weaknesses. In this study, we have primarily used microinjection of previously tested and known constructs which may help mitigate these concerns, and have referenced numerous studies in which these constructs have been used and tested. Nevertheless, the authors are aware of this drawback and have tried to address this previously in other research using novel artificial intelligence techniques (Shen and Lamba et al., 2022 – cited in the manuscript) and this continues to be an active area of investigation for us.

**Reviewer #2 (Public review):**

Summary:

In this study, Lamba and colleagues suggest a molecular mechanism to explain cell heterogeneity in cell specification during pre-implantation development. They show that embryo polarization is asynchronous. They propose that reduced CARM1 activity and upregulation of its substrate BAF155 promote early polarization and trophectoderm specification.

Strengths:

The authors use appropriate and validated methodology to address their scientific questions. They also report excellent live imaging. Most of the data are accompanied by careful quantifications.

Weaknesses:

I think this manuscript requires some more quantification, increased number of embryos in their evaluations and clearly stating the number of embryos evaluated per experiments.

We thank the reviewer for these thoughtful comments on our work, their kind assessment of the strength of our research, and their notes on the weaknesses. We have replied to their points raised below.

Here are some points:

(1) It should be clearly stated in all figure legends and in the text how many cells from how many embryos were analyzed.

We appreciate this comment to provide detailed quantification for every experiment in the paper and stating the numbers of embryos (if a whole embryo level experiment) or blastomeres used for statistical tests and displayed in the graph.

(2) I think that the number of embryos sometimes are too low. These are mouse embryos easily accessible and the methods used are well established in this lab, so the authors should make an effort to have at least 10/15 embryos per experiment. For example "In agreement with this, hybridization chain reaction (HCR) RNA fluorescence in situ hybridization of early 8-cell stage embryos revealed that the number of CDX2 mRNA puncta was higher in polarized blastomeres with a PARD6-positive apical domain than in unpolarized blastomeres, for 5 out of 6 embryos with EP cells (Figure 3A, B)".. or the data for Figure 4, we know how many cells but now how many embryos.

We appreciate the reviewer’s comment regarding the number of embryos used in the hybridization chain reaction (HCR) experiment. We agree that increasing the number of embryos could, in principle, further add statistical power. However, both first authors have since left the lab to begin their postdoctoral training or joining a company, and it is not feasible for us to generate additional embryos at this stage.

Importantly, we believe the number of embryos included in the current manuscript is sufficient to support our conclusions, especially when considered in the context of the broader experimental design, the timing of the study, and our ethical commitment to minimizing animal use.

Notably, the initial HCR experiment targeting Cdx2 mRNA served as a key indication that prompted further investigation of CDX2 at the protein level. These follow-up experiments were conducted with increased numbers of embryos and/or cells and are presented in Figure 3 and the associated supplementary figures (we now have 124 cells (including 23 EP cells) from 16 embryos), thereby strengthening and confirming the conclusion suggested by the HCR data.

(3) It would be useful to see in Figure 4 an example of asymmetric cell division as done for symmetric cell division in panel 4B. This could really help the reader to understand how the authors assessed this.

We used live imaging to track cell division patterns. Cells expressing RFP-tagged polarity proteins were observed during division to identify the resulting daughter cells. Immediately after cytokinesis, we assessed the polarity status of each daughter cell. If both daughter cells were polarized, the division was classified as symmetric; if only one was polarized, it was classified as asymmetric.

**Author response image 1.** 8-cell stage embryos expressing Ezrin-RFP (fire colour) was imaged during 8-16 cell stage division. Top panel arrows indicate a symmetric cell division in which polarity domain became partitioned into both daughter cells; bottom panel indicates asymmetric division in which the polarity domain only get inherited to one cell of the two daughter cells.

(4) Figure 5C there is a big disproportion of the number of EP and LP identified. Could the authors increase the number of embryos quantified and see if they can increase EP numbers?

We thank the reviewer for this comment and want to clarify an important detail: EP cells are a phenomenon with average cellular frequency of less than 10% as compared to LP cells (the other 90%). Therefore, when investigating natural embryo development without bias or exclusion, there will likely be an imbalance in the number of EP and LP cells as is the case for Figure 5C. In this case, morphological differences and clear statistical significance were seen between the shape of EP and LP cells within the cells quantified and therefore we decided not to expend further mice for this particular experiment – but we agree with the comment that in most cases additional embryos would help strength our conclusions further.

(5) Could the authors give more details about how they mount the embryos for live imaging? With agarose or another technique? In which dishes? Overlaid with how much medium and oil? This could help other labs that want to replicate the live imaging in their labs. Also, was it a z-stack analysis? If yes, how many um per stack? Ideally, if they also know the laser power used (at least a range) it would be extremely useful.

We thank the reviewer for this comment and have provided additional detail here and in the Methods section. For live imaging our embryos, we used glass-bottom 35 mm dishes. We then fixed a small cut square of nylon mesh (5mm to 1cm width and height) onto this plate in the centre using silicon which was used as a grid (diameter of approximately 150 micrometres) for deposition of embryos. After drying of the silicon (overnight) and washing with water, the grid was overlaid with a drop of 100 microlitres of KSOM and then covered with mineral oil until this KSOM drop was submerged. After incubation under conditions for live imaging, single embryos were deposited in each ‘well’ of the grid before being placed in the microscope, which was equilibrated at the correct temperature and CO2.
